# Factors Associated with Insomnia Symptoms in a Longitudinal Study among New York City Healthcare Workers during the COVID-19 Pandemic

**DOI:** 10.3390/ijerph18178970

**Published:** 2021-08-26

**Authors:** Marwah Abdalla, Codruta Chiuzan, Yimeng Shang, Gavin Ko, Franchesca Diaz, Kaitlin Shaw, Cara L. McMurry, Diane E. Cannone, Alexandra M. Sullivan, Sung A. J. Lee, Hadiah K. Venner, Ari Shechter

**Affiliations:** 1 Department of Medicine, Columbia University Irving Medical Center, New York, NY 10032, USA; ma2947@cumc.columbia.edu (M.A.); fd2420@cumc.columbia.edu (F.D.); kes2196@cumc.columbia.edu (K.S.); mlc2238@cumc.columbia.edu (C.L.M.); dew2106@cumc.columbia.edu (D.E.C.); as5068@cumc.columbia.edu (A.M.S.); sjl2172@cumc.columbia.edu (S.A.J.L.); hkv2001@cumc.columbia.edu (H.K.V.); 2 Mailman School of Public Health, Columbia University, New York, NY 10032, USA; cc3780@cumc.columbia.edu (C.C.); ys3298@cumc.columbia.edu (Y.S.); wk2343@cumc.columbia.edu (G.K.)

**Keywords:** healthcare worker, insomnia, sleep, COVID-19, mental health

## Abstract

Background: Few studies have examined the longer-term psychological impact of COVID-19 in healthcare workers (HCWs). Purpose: We examined the 10-week trajectory of insomnia symptoms in HCWs during the COVID-19 pandemic. Methods: HCWs completed a web-based survey at baseline (9 April–11 May 2020) and every 2 weeks for 10 weeks. The main outcome was the severity of insomnia symptoms in the past week. Multivariable-adjusted generalized estimating equation analyses examined factors associated with insomnia symptoms. Results: *n* = 230 completed surveys at baseline. *n* = 155, *n* = 130, *n* = 118, *n* = 95, and *n* = 89 completed follow-ups at weeks 2, 4, 6, 8, and 10, respectively. Prevalence of insomnia symptoms of at least moderate severity was 72.6% at baseline, and 63.2%, 44.6%, 40.7%, 34.7%, and 39.3% at weeks 2, 4, 6, 8, and 10, respectively. In multivariable analyses, factors significantly associated with increased odds of insomnia symptoms were younger age (OR: 0.98, 95% CI: 0.96–1.00), working in a COVID-facing environment (OR: 1.75, 95% CI: 1.15–2.67) and hours worked (OR: 1.16, 95% CI: 1.06–1.27). Conclusions: The initial high rates of insomnia symptoms improved as time passed from the peak of local COVID-19 cases but four out of ten HCWs still had moderate-to-severe insomnia symptoms ten weeks after baseline.

## 1. Introduction

During the COVID-19 pandemic, healthcare workers (HCWs) have contended with exposure to the virus and, by extension, risks to their family and friends. These risks and responsibilities confer psychological stress across both professional and personal domains. The magnification of mental and physical harm against the backdrop of the COVID-19 outbreak was called a “parallel pandemic” facing HCWs [1].

HCWs treating COVID-19 patients have experienced acute psychological symptoms, with various meta-analyses reporting high-pooled prevalence for anxiety (23–34%), stress (34–40%) and sleep disturbance (34–64%) [2,3,4]. Much work has been done to document the mental health impact of providing care during the COVID-19 pandemic cross-sectionally, including studies focusing on sleep. For example, two recent meta-analyses found a pooled prevalence of sleep disturbances of 44–45% in HCWs [5,6]. However, we are aware of only two longitudinal studies which examined sleep quality or insomnia symptoms in HCWs during the COVID-19 pandemic [7,8], both from China. Based on the Pittsburgh Sleep Quality Index (PSQI) administered to frontline HCWs in Hubei province during the local peak of the pandemic and ~1 month later, Zhou et al. reported a worsening of sleep quality across the study, from baseline to follow-up, with poor sleep quality (PSQI score > 7) reported in 16.4% of participants at baseline and 27.9% of participants at follow-up [7]. Cai et al. administered the Insomnia Severity Index (ISI) to nurses in Wuhan at two time periods, during the acute outbreak period and ~3–4 weeks later and noted that one-third of participants had symptomatic insomnia which persisted across follow-up [8].

To our knowledge, outside of those reports, no studies have been published on the pandemic’s long-term impact on HCW insomnia symptoms. Insomnia is a cardinal psychological symptom that is linked to the development and progression of psychological disturbance [9]. Furthermore, sleep is a modifiable behavior and therefore a potential target for interventions to decrease psychological distress. We examined the 10-week trajectory of insomnia symptoms in HCWs during the COVID-19 pandemic, with baseline measures collected in Spring of 2020, when New York City was the epicenter of the COVID-19 pandemic in the United States.

## 2. Methods

### 2.1. Study Population and Data Collection

Data are from the longitudinal follow-up of a survey administered to New York City HCWs at a local peak of COVID-19 inpatient admissions [10]. Participants were recruited via emails sent to physicians, advanced practice providers, nurses, and housestaff/fellow listservs at Columbia University Irving Medical Center (CUIMC)/New York Presbyterian Hospital. Baseline surveys were completed between 9 April 2020 and 11 May 2020. A subset of participants from the baseline survey underwent follow-up assessments every 2 weeks for the next 10 weeks. The CUIMC Institutional Review Board approved all procedures. All participants provided electronic informed consent.

The survey included questions on demographics (age, sex, race, ethnicity), clinical role, clinical setting during the local peak of COVID-19, and number of working hours in the past week. The main outcome of interest in the current report was presence of insomnia symptoms that was assessed with the following question: “Over the past week, what is the severity of any insomnia symptoms you experienced (e.g., poor quality sleep, difficulty falling asleep or staying asleep, waking up too early, feeling that sleep is not refreshing)?” The answer options were “(0) none”, “(1) mild”, “(2) moderate”, “(3) severe” and “(4) very severe”. When our study was being designed and conducted in April–May 2020, New York City was in the midst of the local peak in COVID-19 patient admissions and was then the global epicenter of COVID-19 cases. Our goal was to collect data in a manner that was minimally burdensome to HCWs on the frontlines and to reduce any interruption of clinical care during the pandemic where possible. We therefore specifically designed a rapid sleep assessment as part of a larger questionnaire. The current single-item sleep assessment is based on the ISI and was designed to be consistent with the “insomnia problem” section of the ISI (i.e., items 1–3) which measures difficulty falling asleep, difficulty staying asleep, and problems waking up too early with a 5-point Likert scale to rate the severity (i.e., none; mild; moderate; severe; very severe) [11].

### 2.2. Statistical Analysis

Descriptive statistics are summarized as frequencies (percentages) for the categorical variables and median (inter-quartile range, IQR) for age and number of hours worked in the past week. Based on the clinical relevance and frequency distributions, the main outcome of the analysis was categorized into two groups: “insomnia symptoms of at least moderate severity” (≥2) vs. “mild or no symptoms” (<2). Participant’s race/ethnicity was also dichotomized into “White/Non-Hispanic or Latino” vs. “Other”.

We calculated the prevalence of a positive screen for insomnia symptoms (rating of moderate, severe, or very severe) at each timepoint. To investigate the factors associated with presence of insomnia symptoms of at least moderate severity, a generalized estimating equation (GEE) model was applied to accommodate the correlated data of repeated measurements (at baseline and at 2, 4, 6, 8, 10 weeks follow-up) with a logit link function and an exchangeable correlation structure. We conducted both univariable and multivariable GEE analyses using the following pre-selected covariates recorded at baseline: age, sex (female vs. male), race/ethnicity (White, non-Hispanic/Latino vs. other), clinical role (registered nurse [RN] vs. other), clinical setting during the local peak of COVID-19 (working in a COVID-19-focused area, defined as the emergency department, intensive care unit, inpatient or outpatient COVID-19 areas vs. working in a non-COVID-19 area), and overall total number of hours worked over the past week with categories ranging from (1) 0–10 h to (13) more than 120 h. In order to achieve balance between the groups (see Table 1), we combined “White/Non-Hispanic or Latino” vs. “Other” and also compared RN vs. Other for the purposes of the multivariable analyses. The goodness of fit of the multivariable model was assessed using an extension of the Hosmer and Lemeshow statistic for repeated binary observations, using predicted deciles of risk [12]. For the GEE multivariable model, three working correlation structures (unstructured, exchangeable, and auto-regressive 1 AR (1)) were tested and compared based on QIC (complexity) and goodness of fit (based on Hosmer-Lemeshow statistic). All statistical analyses were performed in R v.4.1.0 (R Foundation for Statistical Computing, Vienna, Austria) using a two-sided type I error of 0.05.

## 3. Results

Of the *n* = 827 in the original cross-sectional survey, *n* = 230 (27.8%) agreed to participate in the 10-week longitudinal follow-up; this cohort represented the study group considered in the analysis. Participants in the current report were predominantly women (79.6%), white (64.3%), and not Hispanic or Latino (86.8%). The majority of participants were nurses (50.0%), worked in a COVID-facing setting (82.6%), and worked 41–50 h during the local peak week (Table 1).

In order to assess participation bias, we compared the two cohorts: those participants from the original cross-sectional study who agreed to participate in the longitudinal study (*n* = 230) and those who did not agree to participate in the longitudinal study (*n* = 597). The two groups were similar with respect to demographics (i.e., age, sex, race, ethnicity), work-related factors (i.e., clinical location, hours worked, clinical role), and insomnia severity at baseline. In the current report of the *n* = 230 at baseline, *n* = 155, *n* = 130, *n* = 118, *n* = 95, and *n* = 89 participants completed follow-ups at weeks 2, 4, 6, 8, and 10, respectively. We conducted a comparison of baseline participant characteristics among those who completed the 10-week follow-up assessment (*n* = 89) and those who did not complete the 10-week follow-up assessment (*n* = 141) (Table 2). There were differences in age (younger in those who completed the 10-week follow-up vs. those who did not complete the follow-up), clinical location (higher proportion were COVID-facing in the completers vs. non-completers groups), and role (higher proportion of registered nurse in the completers vs. non-completers groups). All of these variables were included in the multivariable GEE model.

**Figure 1:**
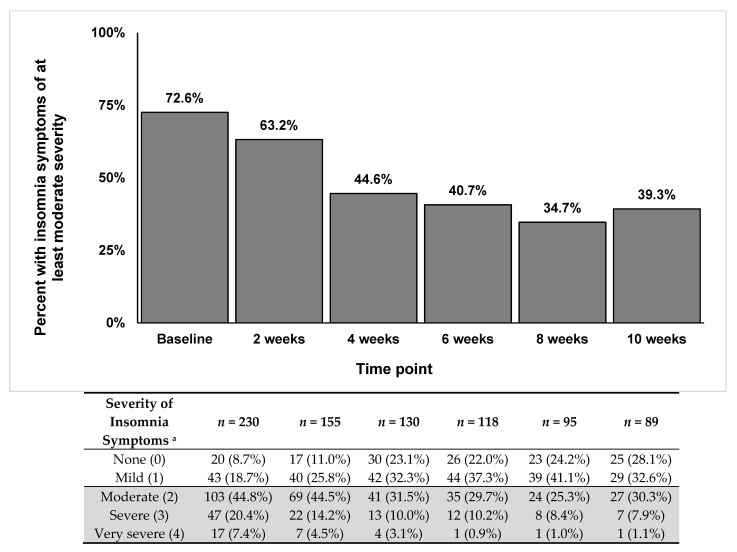
Prevalence of participants reporting insomnia symptoms of at least moderate severity across the 10-week follow-up period.; ^a^ Ratings were on a 0–4 scale (none, mild, moderate, severe, very severe) for the following question: “Over the past week, what is the severity of any insomnia symptoms you experienced (e.g., poor quality sleep, difficulty falling asleep or staying asleep, waking up too early, feeling that sleep is not refreshing)?”.

The prevalence of at least moderate insomnia symptoms was 72.6% at baseline, 63.2% at week 2, 44.6% at week 4, 40.7% at week 6, 34.7% at week 8, and 39.3% at the 10-week follow-up. Figure 1 shows the steady decrease of the presence of moderate-to-severe symptoms over time, while the percentages of mild or no symptoms increased across the 10-week follow-up period.

In the GEE univariable analyses, factors that were significantly associated with increased odds of insomnia symptoms were younger age (odds ratio [OR]: 0.97, 95% CI: 0.96–0.99, *p* = 0.005; age was treated as a continuous variable so for a 1-y increase in age, the odds of developing insomnia of at least moderate severity decrease by 3%), female sex (OR: 1.75, 95% CI: 1.03–3.00, *p* = 0.039), working in a COVID-facing environment (OR: 1.95, 95% CI: 1.31–2.94, *p* = 0.001) and amount of hours worked during the COVID-19 peak (OR: 1.14, 95% CI: 1.05–1.25, *p* = 0.003). In the multivariable analyses, factors that remained significant were younger age (OR: 0.98, 95% CI: 0.96–1.00, *p* = 0.031), working in a COVID-facing environment (OR: 1.75, 95% CI: 1.15–2.67, *p* = 0.008), and the number of hours (10 h increments) worked during the COVID-19 peak (OR: 1.16, 95% CI: 1.06–1.27, *p* = 0.002) (Table 3). The exchangeable structure applied to the GEE multivariable model generated the lowest QIC (1013.28) and a *p*-value = 0.26 for the overall (Hosmer-Lemeshow) goodness of fit, indicating that the selected model was a good fit for the data.

## 4. Discussion

To our knowledge, this is the first longitudinal examination of insomnia symptoms in HCWs in the United States during the COVID-19 pandemic. We report that the initial high rates of insomnia symptoms improved as more time passed from the initial peak of local COVID-19 cases, but remained elevated 10 weeks after baseline.

We observed that higher working hours were associated with increased odds of insomnia symptoms and this effect increased with longer hours. For example, participants who worked between 61–70 h had 185% higher odds of insomnia symptoms compared to those who worked only 0–10 h per week. Working in a COVID-facing location was also associated with increased odds of insomnia symptoms in multivariable analyses. These are consistent with prior findings of the adverse psychological toll, including heightened anxiety and insomnia, that was also reported for HCWs during the Middle East Respiratory Syndrome (MERS) [13] and Severe Acute Respiratory Syndrome (SARS) [14,15,16] outbreaks. Specifically, in a systematic review of the occupational factors and the psychological outcomes in HCWs during infectious disease outbreaks, working in a high-risk environment and in more direct patient care were among the most important factors related to poor mental health outcomes, including poor sleep [17]. Research on the SARS outbreak also indicates that HCWs with a greater exposure to infected patients saw sustained higher levels of distress and posttraumatic stress 1–2 years later [18]. These findings are relevant for understanding psychological risk in HCWs during the COVID pandemic and suggest that psychological risk varies by degree of exposure. Our current observations are also in alignment with established theories of insomnia, as higher hours worked during COVID and more time spent in COVID-facing environments directly engaging with patients are likely to contribute to precipitation and perpetuation of insomnia symptoms [19].

Our main study findings are in contrast to the findings in the study by Zhou et al. which assessed sleep quality using the Pittsburgh Sleep Quality Index questionnaire among 494 healthcare workers in Hubei province, China at baseline during the COVID-19 outbreak (21 February–6 March 2020) and again after 4–6 weeks [7]. Zhou et al. demonstrated that poor sleep quality, as defined with the PSQI, increased from 16.4% at baseline assessment to 27.9% during the follow-up period. An important methodological difference between the study by Zhou et al. and our current study exists. Participants in our study were asked to assess their current sleep disturbances i.e., the presence of insomnia symptoms over the past week, and underwent follow-up assessments every two weeks, rather than a one-time repeat assessment which was performed in the study by Zhou et al. In the study by Zhou et al., participants were asked to assess their sleep quality over the past month (via the PSQI) and therefore the follow-up assessment may have reflected participants’ sleep status during and immediately following the acute phase of the COVID-19 pandemic in Hubei province rather than a true assessment of their current sleep symptoms in the weeks after the COVID-19 outbreak subsided in Hubei province.

While our study did not assess individual reasons for changes in sleep difficulties across time for participants in the study, we hypothesize that there may be several possible reasons why there was a lower prevalence of moderate-to-severe insomnia symptoms at 10 weeks compared to baseline. Our baseline study was conducted at the height of the first wave of the COVID-19 pandemic in New York City (April–May 2020). COVID-19 cases in New York City began to decrease in June–July 2020 and stay-at-home orders were lifted as the city entered the first phase of reopening, the timing of which coincided with the follow-up period of our study. Because of the decrease in hospitalized patients with COVID-19, the redeployment of some HCWs was no longer needed, allowing some of these individuals to remain in their “own-occupation” clinical settings. As such, some participants were no longer working in COVID-19 facing settings. Similarly, clinical work hours may also have decreased for some participants. Lastly, several well-being and mental health programs were also created during this follow-up period including at our institution, regionally, and nationally. HCWs who may have participated in well-being programs may have experienced secondary improvement in sleep disturbances as their overall well-being was addressed.

Several current limitations are worth noting. This was a single-center study, conducted in a large medical center in an urban environment, which may limit the generalizability of the findings. This study had a relatively short follow-up, although it is to our knowledge the longest conducted to date. We also used a single-item question to capture broad insomnia symptoms, as opposed to a more formal or validated questionnaire for assessment (e.g., the ISI). The single-item question to assess insomnia symptom severity that was used here was based on the ISI and captures many of the same symptoms (difficulty falling or staying asleep, waking up to early) on the same severity scale (none to very severe). However, aspects of the item such as “feeling that sleep is unrefreshing” may be due to other sleep disorders which were not assessed in this study. It is possible that the use of this single-item questionnaire could have impacted the results. The non-validated item we used may have relatively lower specificity than other established questionnaires, thereby contributing to an over-estimation of insomnia symptoms. Another limitation is the sample attrition, as more than half of the sample was lost by the final follow-up assessment at 10 weeks following the initial assessment. A larger sample size would also allow for more nuanced subgroup analyses, e.g., within nurses or physicians separately. Finally, the self-selected cohort might be subject to participation/self-selection bias.

## 5. Conclusions

Many hospital systems have implemented mental health initiatives in response to the pandemic. Our findings suggest that these initiatives should be continued. Personalized interventions could be tailored to individual COVID-related work burden. Targeted interventions to improve sleep in HCWs in the wake of the pandemic may also benefit HCWs’ overall mental health [20].

## Figures and Tables

**Table 1:** Baseline characteristics of participants who agreed to participate in the longitudinal follow-up assessments (*n* = 230).

	(*n* = 230)
Age (years), median (IQR)	36 (31–48)
Sex, *n* (%)	
Female	183 (79.6%)
Male	46 (20.0%)
Other	1 (0.4%)
Race, *n* (%)	
White	148 (64.3%)
Asian	26 (11.3%)
Black	21 (9.1%)
Other	20 (8.7%)
More than one race	14 (6.1%)
Hawaiian/Pacific Islander	1 (0.4%)
American Indian/Native American	0 (0%)
Ethnicity, *n* (%)	
Not Hispanic or Latino	191 (86.8%)
Hispanic or Latino	29 (13.2%)
Clinical location, *n* (%)	
COVID-facing	190 (82.6%)
Not COVID-facing	40 (17.4%)
Hours worked in past week (at baseline) ^a^	
Median (IQR)	41–50 h (31–40 h, 51–60 h)
Role, *n* (%)	
Registered Nurse	115 (50.0%)
Attending Physician	50 (21.7%)
Resident	31 (13.5%)
Advanced Practice Provider	13 (5.7%)
Fellow	12 (5.2%)
Other	8 (3.5%)
Prefer not to answer	1 (0.4%)
Dichotomized insomnia severity, *n* (%)	
Moderate or severe symptoms (≥2 score)	167 (72.6%)
None or mild symptoms (<2 score)	63 (27.4%)

^a^ Selections were based on 13 categories: 0–10 h, 11–20 h, 21–30 h, 31–40 h, 41–50 h, 51–60 h, 61–70 h, 71–80 h, 81–90 h, 91–100 h, 101–110 h, 111–120 h, 120 + h.

**Table 2:** Comparison of baseline characteristics between participants who completed the 10-week follow-up assessment (*n* = 89) and those who did not complete the 10-week follow-up assessment (*n* = 141).

	Completed 10-Week Assessment (*n* = 89)	Did Not Complete 10-Week Assessment (*n* = 141)	*p*-Value ^c^
Age (years), median (IQR)	35 (31–44.3)	41 (32–53)	**0.004**
Sex (*n*%)			0.408
Female	114 (80.9%)	69 (77.5%)	
Male	27 (19.1%)	19 (21.3%)	
Other	0 (0%)	1 (1.1%)	
Race (*n*%)			0.334
White	84 (59.6%)	64 (71.9%)	
Asian	16 (11.3%)	10 (11.2%)	
Black	14 (9.9%)	7 (7.9%)	
Other	16 (8.7%)	4 (4.5%)	
More than one race	10 (7.1%)	4 (4.5%)	
Hawaiian/Pacific Islander	1 (0.7%)	0 (0%)	
American Indian/Native American	0 (%)	0 (0%)	
Ethnicity (*n*%)			0.302
Not Hispanic or Latino	118 (83.7%)	73 (82%)	
Hispanic or Latino	23 (16.3%)	16 (18%)	
Clinical location (*n*%)			**0.020**
COVID-facing	123 (87.2%)	67 (75.3%)	
Not COVID-facing	18 (12.8%)	22 (24.7%)	
Hours worked in past week (at baseline) ^a^			0.348
Median (IQR)	41–50 h (31–40 h, 51–60 h)	41–50 h (31–40 h, 51–60 h)	
Role (*n* %)			**0.009**
Registered Nurse	78 (55.3%)	37 (41.6%)	
Attending Physician	19 (13.5%)	30 (33.7%)	
Resident	23 (16.3%)	8 (9%)	
Advanced Practice Provider	0 (0%)	1 (1.1%)	
Fellow	9 (6.4%)	3 (3.4%)	
Other	11 (7.8%)	10 (11.2%)	
Prefer not to answer	1 (0.7%)	0 (0%)	
Severity of insomnia symptoms ^b^			0.160
Median (IQR)	2.00 (2.00, 3.00)	2.00 (1.00, 3.00)	
Dichotomized insomnia severity			0.088
None or mild symptoms (<2 score)	33 (23.4%)	30 (33.7%)	
Moderate or severe symptoms (≥2 score)	108 (76.6 %)	59 (66.3%)	

^a^ Selections were based on 13 categories: 0–10 h, 11–20 h, 21–30 h, 31–40 h, 41–50 h, 51–60 h, 61–70 h, 71–80 h, 81–90 h, 91–100 h, 101–110 h, 111–120 h, 120 + h; ^b^ Ratings were based on a 0–4 scale (none, mild, moderate, severe, very severe) for the following question: “Over the past week, what is the severity of any insomnia symptoms you experienced (e.g., poor quality sleep, difficulty falling asleep or staying asleep, waking up too early, feeling that sleep is not refreshing)?”; ^c^ *p*-values are based on chi-squared/Fisher Exact test for categorical variables and Wilcoxon Rank-Sum test for continuous variables. *p*-values in bold indicate statistically significant differences (*p* < 0.05).

**Table 3:** Generalized estimating equation (GEE) univariable and multivariable models for presence of insomnia symptoms from baseline to 10 weeks follow-up.

	Univariable Model			Multivariable Model		
Variable	B (SE)	OR (95% CI)	*p*-Value	B (SE)	OR (95% CI)	*p*-Value
Age	−0.03 (0.01)	0.97 (0.96, 0.99)	**0.005**	−0.02 (0.01)	0.98 (0.96, 1.00)	**0.031**
Role (RN vs. other)	0.32 (0.21)	1.38 (0.91, 2.10)	0.135	0.34 (0.23)	1.40 (0.89, 2.22)	0.141
Sex (female vs. male)	0.56 (0.27)	1.75 (1.03, 3.00)	**0.039**	0.53 (0.29)	1.70 (0.96, 3.02)	0.069
Clinical location (COVID-facing vs. not)	0.67 (0.21)	1.95 (1.31, 2.94)	**0.001**	0.56 (0.21)	1.75 (1.15, 2.67)	**0.008**
Work hours ^a^	0.13 (0.04)	1.14 (1.05, 1.25)	**0.003**	0.15 (0.05)	1.16 (1.06, 1.27)	**0.002**
Race/ethnicity (White, Non-Hispanic/Latino vs. other)	−0.25 (0.22)	0.78 (0.51, 1.20)	0.257	−0.14 (0.23)	0.87 (0.56, 1.36)	0.536

B (SE): regression coefficient and standard error, OR (95% CI): odds ratio and 95% confidence interval, RN: registered nurse, SE: standard error.; ^a^ Coefficients and ORs for “Work hours” were calculated for 10 h increments across the categories: 0–10 h, 11–20 h, 21–30 h, 31–40 h, 41–50 h, 51–60 h, 61–70 h, 71–80 h, 81–90 h, 91–100 h, 101–110 h, 111–120 h, 120+ h. *p*-values in bold indicate statistical significance (*p* < 0.05).

## Data Availability

The data presented in this study are available on request to the corresponding author.
